# Youths’ awareness and attitudes towards raising the minimum legal age of smoking and passive smoking in Singapore

**DOI:** 10.3389/fpubh.2024.1359929

**Published:** 2024-07-11

**Authors:** Kalaipriya Gunasekaran, Prashwin Singh, Ding Xuan Ng, Eileen Yi Ling Koh, Huan Yu Lee, Rei Tan, Yier Wang, Ngiap Chuan Tan

**Affiliations:** ^1^Department of Research, SingHealth Polyclinics, Singapore, Singapore; ^2^Faculty of Medicine, Universiti Malaya, Kuala Lumpur, Malaysia; ^3^Hwa Chong Institution, Singapore, Singapore; ^4^SingHealth-Duke NUS Family Medicine Academic Clinical Program, Singapore, Singapore

**Keywords:** cessation, national health policy, passive smoking, smoking, youth

## Abstract

**Objectives:**

Early smoking initiation has been associated with a higher risk of developing long-term smoking habit. There is a growing global consensus that demands raising the minimum legal age (MLA) for smoking as an approach to address this problem. Singapore successfully raised the MLA from 18 to 21 years in 2021. This study aimed to evaluate the awareness and attitude of multi-ethnic Asian youth (aged 15–24) on raising MLA to 21 and passive smoking.

**Methods:**

A cross-sectional survey comprising of 23 items was circulated via a secure internet-based platform, FORMSG between September and November 2022. Data were analyzed for descriptive statistics. Categorical variables were compared for association with receptivity toward change in MLA using Chi-Squared test and multivariable logistic regression analysis using Rstudio. Post-hoc Bonferroni correction were further utilized for pairwise comparison.

**Results:**

Majority (80.3%) of the 608 participants expressed their support for MLA 21 implementation. Participants’ age was a significant variable as those aged 15–17 years old (OR = 2.1, 95%CI = 1.01–4.32, *p* = 0.048) showed a higher likelihood of supporting MLA implementation compared to those aged 21 and above. In addition, majority (89.8%) of them were also aware of the harmful effects of passive smoking. When it came to discouraging smoking among youth, family influence (64%) and school education (55.6%) emerged as the top strategies.

**Conclusion:**

Most of the youth express strong support for raising the MLA to 21, with over 80% in favor of such change, reflects a significant harmony among youth in favor of tobacco-free environment.

## 1 Introduction

Smoking is a major risk factor for cardiovascular, airway diseases, reproductive abnormalities, cancer, and disruption of the immune system (1). A staggering 7.69 million deaths were attributable to smoking and resulted in loss of 200 million Disability Adjusted Life Years (2). However, those who quit smoking between the ages 15 to 34 years had about the same mortality risk from major cardiovascular diseases as their peers of similar ages (3).

In 2019, there were approximately 1.14 billion tobacco smokers globally, of which an estimated 155 million of them are between 15 and 24 years of age (2). The majority (83%) of them initiated smoking when they were 14–25 years old (4). In Singapore, there has been a reduction in the prevalence of smoking from 11.8% in 2017 to 10.1% in 2020. This decline can be attributed to stringent regulations, including increased cigarette taxes, enhanced package warnings, and the raising of the legal smoking age to 21. These measures have also led to decreased smoking among young adults aged 18–29 (5).

The initiation of smoking before the age of 21 is a prevalent trend in Singapore, with 95% of smokers beginning their habit during adolescence (6). Smoking initiation during early teenage years leads to more extensive cigarette use and increased difficulty in its cessation (7). The tobacco-induced neurotoxicity of adolescent cognitive development (TINACD) model alludes to its detrimental effect on the actively developing prefrontal cortex in youth, up to their mid-20s, which may result in their poor impulse control in life (8).

The World Health Organization (WHO) Framework Convention on Tobacco Control ratified by 180 parties requires the prohibition of sales of tobacco products to or by persons under 18 years of age (9). The rationale of such restrictions includes reducing the prevalence and early onset of smoking. In England and Wales, raising the minimum legal age (MLA) of smoking from 16 to 18 years translated into the greatest percentage decrease of smoking among those aged 16–17 years (10).

More recently, public health and policy makers have been advocating to raise the MLA of smoking to 21 years. The US Institute of Medicine in 2015 reported that such a measure would lead to a 25% decline in smoking initiation among youth aged 15–17 years, 50,000 fewer cases of lung cancer and prevent 223,000 early deaths in the US (11).

MLA 21 laws have already been implemented in 14 states and more than 450 cities and counties in the US (12). Needham, Massachusetts, pioneered the Tobacco 21 movement and reported greater declining smoking rate compared to its surrounding communities (10). Likewise, Singapore at the center of Southeast Asia has enacted similar laws gradually, raising the MLA from 18 years in 2018 to 21 years in 2021 (13). The decline in smoking from 9.8% in 2017 to 8.8% in 2020 among youth and young adults aged 18–29 in Singapore has been attributed to raising the MLA (14).

Nevertheless, local data has reported that 7 in 1,000 students from primary to high schools were guilty of smoking and vaping in 2022 (15). Critics have now questioned the efficacy of MLA 21 laws. Youth and young adults may perceive the age restriction to be merely a “rite of passage.” MLA 21 laws may imply that smoking is acceptable from age 21 onwards, further normalizing the link between smoking and adulthood (16). Besides, MLA 21 laws do not eliminate the possibility of youth procuring cigarettes from their older friends, family or even strangers (17).

As an alternative to MLA21 laws, the tobacco-free generation proposal champions the transition from tobacco control to a tobacco free future (18). In 2022, New Zealand became the first country to pass a tobacco-free generation law prohibiting the sale of tobacco to anyone born on and after 2009 (19). Khoo et al. proposed a similar Singaporean model of the tobacco-free generation by denying access to tobacco for individuals born after 2000 (16). A survey conducted among Singaporeans in 2007 indicated that approximately 70% of Singaporeans would support such a model (16).

The adverse effects of smoking impact on passive smokers who inhale environmental tobacco smoke (ETS) released by burning cigarettes. Evidence has emerged to reveal the association between passive smoking and lung cancer, invasive meningococcal disease, and allergic disease (20). Singaporeans, both young and old, are at high risk for passive smoking due to proximity between multi-unit residential areas which are inhabited by 80% of Singaporeans, allowing for ETS to drift into neighboring homes (21). In accordance with the Global Burden of Disease 2019 study, the annual mortality rates attributed to second-hand smoke exposure in Singapore was estimated at 261 in 2015, increasing steadily to 296 in 2019 (22).

Local youth are the target population of the national smoking prohibition policies on the densely populated island state regardless of their smoking status. Their voices and perspectives on these deterrent policies provide insights to the degree of adoption of the MLA 21.

### 1.1 Aim

This study aimed to assess the views of Singaporean youth (aged 15–24) on the MLA21 laws and pre-existing smoking prevention measures. It also aimed to assess their awareness on passive smoking and its associated harms.

The findings would indicate their receptivity toward MLA21 laws and signal their potential support for stricter tobacco regulation. Given the importance of engaging youth in policy making (23), these findings could potentially allow their valuable views to be considered in the legislative process for tobacco control.

## 2 Methods

A cross-sectional questionnaire study was conducted electronically among youth in Singapore, where 11.4% of the local population comprise youth aged from 15 to 24 years (24, 25). Convenience sampling technique was employed to recruit participants through distributing the e-questionnaire via social media network such as WhatsApp™ chat groups and Telegram™ channels from September to November 2022 (26). This would help to mitigate potential accuracy concern on participants’ response, given the possibility that some may be minors for whom smoking is illegal. The purpose, anonymity and voluntary participatory process of the e-survey was conveyed to the potential participants based on digitalized standardized information sheet and participation implied consent.

### 2.1 Eligibility criteria

Multi-ethnic Asian youth aged between 15 and 24 years old were included.

### 2.2 Sample size estimation

According to Dai (27), the proportion of youth supporting Tobacco 21 was 63.9%. Using 99% confidence level and 5% precision, the minimum sample size required is 612 using sample size for proportion. With an estimated average response rate of 35% for internet survey, the survey should be sent out to at least 1749 youth (28, 29).

### 2.3 Data collection and management

Data from the questionnaire, collected in .pst file format, was collated before converting to excel file format through the Data Collation Tool provided by FORMSG. A data analyst ensured survey completion and the data was then stored in a password protected database and was made accessible only to the study team members.

### 2.4 Questionnaire

Established and validated questionnaire specifically to seek responses for MLA 21 and measures to curb smoking was not available. A self-developed 23-item questionnaire comprising of four sections guided and co-created by an experienced clinician (TNC) was prepared covering these domains:

- Demographics and smoking status
  Data was collected on age, sex, ethnicity, highest education level and smoking status from the participants.
- Effectiveness and support on MLA21
  Participants would provide data on their perceived effectiveness of MLA21 and raising the age as a deterrent against smoking.
- Effectiveness of smoking prevention measures
  Participants were asked about the factors associated with smoking and to identify their perceived effective measures to eradicate smoking.
- Awareness and experience on passive smoking
  Data was collected on participants’ awareness on passive smoking, its effects on their health, and exposure to secondary smoke.

Participants’ support toward the MLA 21 bill was measured from the question “What are your views on raising the minimum legal age of smoking to 21 years old?” under the section “Effectiveness and support on MLA 21.” Participants who responded Strongly Supportive and Supportive were categorized as those supportive of the MLA 21 bill. The Cronbach’s alpha value was computed to assess the internal consistency of participants’ perception toward effectiveness of smoking preventive measure. The obtained coefficient of α=0.76 indicated an acceptable internal consistency.

The validity of the questionnaire was assessed by 3 eligible youths. It aims to evaluate their understanding on the clarity of the questionnaire and a 100% affirmative response rate was obtained.

### 2.5 Statistical analyses

Data were presented in frequencies and percentages for categorical demographics. Categorical parameters were compared for association with receptivity toward change in MLA using Chi-Squared test. Factors that were significant (*p* ≤ 0.2) from bivariate analysis were included in the multivariable logistics regression model to account for potential confounders. Odds ratio and their confidence interval were presented for factors associated with support toward the MLA change. Comparisons were also made across multiple age groups to detect any significant difference on youth’s view for the effectiveness of preventive measures. Post-hoc Bonferroni correction were further utilized for pairwise comparison. All analysis was carried out R version 3.5.2, Rstudio and IBM SPSS Statistics Version 26. Statistical significance was set at *p* ≤ 0.05. Descriptive statistics were calculated in the form of frequencies and percentages to describe awareness and sources of passive smoking, effectiveness of measures to discourage smoking and measures rated by participants to discourage smoking.

## 3 Results

### 3.1 Demographics and views on MLA

A total of 1926 surveys was distributed out and 608 participants completed the questionnaire (response rate of 31.6%). Table 1 summarizes the demographics of participants. Majority of participants were aged 15–17 years (68.3%), female (56.7%), Chinese ethnicity (93.8%), had qualifications above secondary education (55.8%) and were non-smokers (96.7%) and had experience of indoor passive smoking (78%).

**Table 1:** Demographic characteristics of youth for change in smoking minimum legal age (MLA).

Characteristics	Total (%)	Supportive of MLA (%)	Not Supportive of MLA (%)	*p*-value
Total	608 (100)	488 (80.3)	120 (19.7)	
Age (years)				0.005
15–17	415 (68.3)	342 (82.4)	73 (17.6)	
18–20	148 (24.3)	118 (79.7)	30 (20.3)	
21–24	45 (7.4)	28 (62.2)	17 (37.8)	
Gender				0.061
Female	345 (56.7)	286 (82.9)	59 (17.1)	
Male	263 (43.3)	202 (76.8)	61 (23.2)	
Ethnicity				0.021
Chinese	570 (93.8)	463 (81.2)	107 (18.8)	
Others	38 (6.2)	25 (65.8)	13 (34.2)	
Highest qualifications				0.097
Up to secondary	269 (44.2)	224 (83.3)	45 (16.7)	
Post-secondary and above	339 (55.8)	264 (77.9)	75 (22.1)	
Smoking status				< 0.001
Non-smoker	588 (96.7)	479 (81.5)	109 (18.5)	
Smoker/Past smoker	20 (3.3)	9 (45)	11 (55)	
Experienced indoor passive smoking				0.722
No	134 (22)	109 (81.3)	25 (18.7)	
Yes	474 (78)	379 (80)	95 (20)	

Most youth were supportive of the MLA 21 bill (80.3%). Age (*p* = 0.005), ethnicity (*p* = 0.021) and smoking status (*p* < 0.001) were significantly associated with support toward the MLA 21 bill. Younger youth aged 15–17 were more supportive of MLA bill (82.4%) compared to older youth aged 21–24 (62.2%). Chinese participants were also more supportive of the MLA bill (81.2%). Furthermore, participants who have never smoked were more supportive of the MLA bill (81.5%).

Table 2 shows the factors associated with support for MLA 21 bill. Multivariable logistic regression revealed that participants aged 15–17 years old (OR = 2.1, 95%CI = 1.01–4.32, *p* = 0.048) and those who have never smoked (OR = 4, 95%CI = 1.5–10.81, *p* = 0.005) were more likely in support of the MLA 21 bill. Factors of gender, ethnicity and educational qualifications were not significantly associated with support for the MLA 21 bill.

**Table 2:** Factors associated with support for MLA 21 bill using logistic regression.

	Odds ratio (95%CI)	*P*-value
Age		
15–17	2.1 (1.01–4.32)	0.048
18–20	1.91 (0.87–4.08)	0.099
21–24	Ref	–
Gender		
Female	Ref	–
Male	0.67 (0.45–1.01)	0.059
Ethnicity		
Chinese	1.56 (0.69–3.33)	0.262
Others	Ref	–
Highest qualifications		
Up to secondary	Ref	–
Secondary and beyond	0.78 (0.5–1.23)	0.291
Smoking status		
Smoker/Past-smoker	Ref	–
Non-smoker	4 (1.5–10.81)	0.005

Table 3 reports youths’ views on smoking preventive measures. School education (*p* < 0.001), family influence (*p* = 0.006) and cigarette pricing (*p* < 0.001) significantly differ across age groups. Bonferroni-corrected post-hoc test revealed that a larger proportion of younger youth aged 15–17 years (p < 0.001) and 18–20 years (*p* = 0.014) viewed school education as effective in discouraging smoking. More younger youth aged 15–17 years (*p* < 0.001) and 18–20 years (*p* = 0.003) also viewed price of cigarette to be important to discourage smoking. However, significantly more youth aged 15–17 years (16.6%) perceived family influence as an encouragement to smoke compared to those aged 21–24 years (2.2%) (*p* = 0.032).

**Table 3:** Difference in views of smoking preventive measures across age group.

	Total	15–17 years	18–20 years	21–24 years	*P*-value
School education					< 0.001
Discourage	543 (89.3)	380 (91.6)	131 (88.5)	32 (71.1)	
Neutral	59 (9.7)	30 (7.2)	16 (10.8)	13 (28.9)	
Encourage	6 (1)	5 (1.2)	1 (0.7)	0 (0)	
Smoking area restriction					0.925
Discourage	469 (77.1)	318 (76.6)	117 (79.1)	34 (75.6)	
Neutral	120 (19.7)	84 (20.2)	27 (18.2)	9 (20)	
Encourage	19 (3.1)	13 (3.1)	4 (2.7)	2 (4.4)	
Family influence					0.006
Discourage	429 (70.6)	289 (69.6)	109 (73.6)	31 (68.9)	
Neutral	94 (15.5)	57 (13.7)	24 (16.2)	13 (28.9)	
Encourage	85 (14)	69 (16.6)	15 (10.1)	1 (2.2)	
Peer influence					0.078
Discourage	255 (41.9)	170 (41)	63 (42.6)	22 (48.9)	
Neutral	127 (20.9)	77 (18.6)	38 (25.7)	12 (26.7)	
Encourage	226 (37.2)	168 (40.5)	47 (31.8)	11 (24.4)	
Anti-smoking advertisements					0.755
Discourage	459 (75.5)	316 (76.1)	112 (75.7)	31 (68.9)	
Neutral	139 (22.9)	92 (22.2)	34 (23)	13 (28.9)	
Encourage	10 (1.6)	7 (1.7)	2 (1.4)	1 (2.2)	
Image packaging					0.326
Discourage	432 (71.1)	292 (70.4)	106 (71.6)	34 (75.6)	
Neutral	160 (26.3)	113 (27.2)	39 (26.4)	8 (17.8)	
Encourage	16 (2.6)	10 (2.4)	3 (2)	3 (6.7)	
Cigarette price					< 0.001
Discourage	506 (83.2)	364 (87.7)	113 (76.4)	29 (64.4)	
Neutral	88 (14.5)	44 (10.6)	31 (20.9)	13 (28.9)	
Encourage	14 (2.3)	7 (1.7)	4 (2.7)	3 (6.7)	
Smoking alternative					0.225
Discourage	266 (43.8)	185 (44.6)	61 (41.2)	20 (44.4)	
Neutral	96 (15.8)	56 (13.5)	32 (21.6)	8 (17.8)	
Encourage	246 (40.5)	174 (41.9)	55 (37.2)	17 (37.8)	

**Figure 1:**
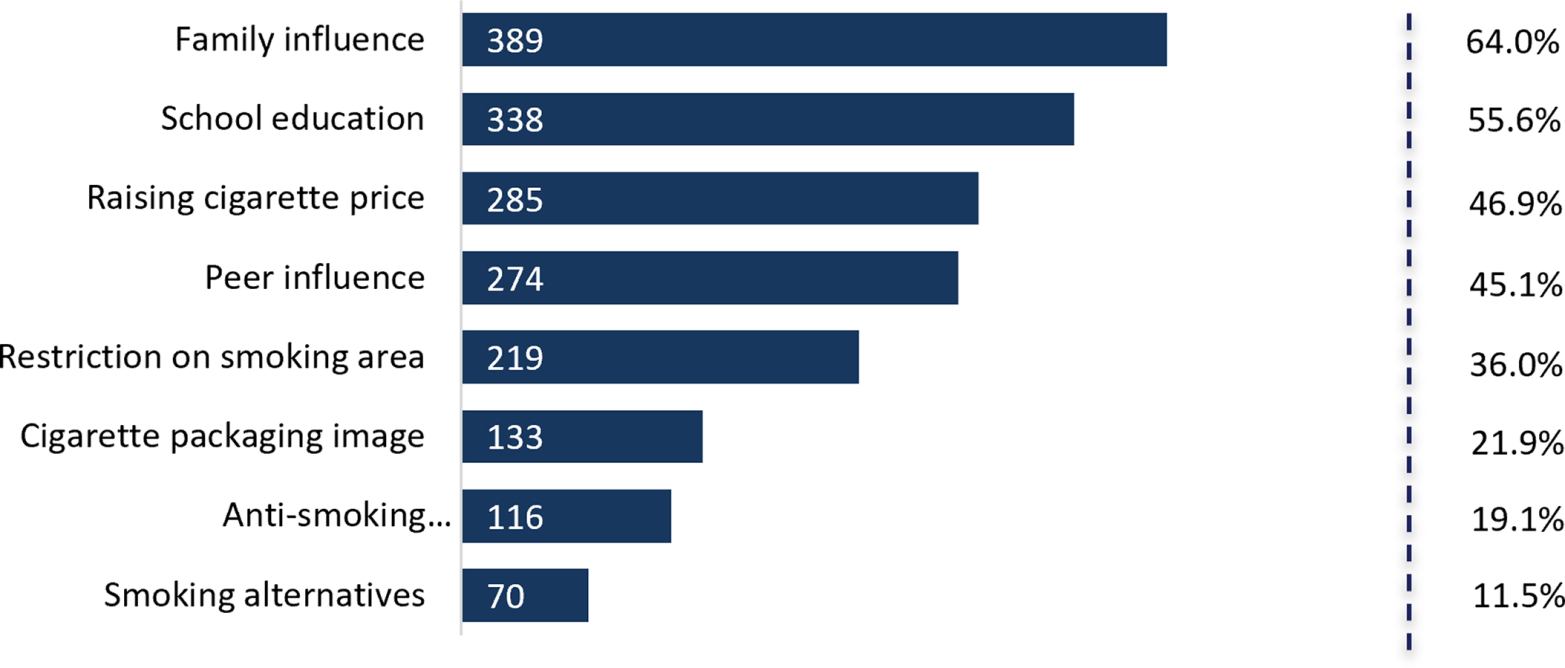
Measures to effectively discourage youth from smoking.

Figure 1 reflects the views of youth on the measures to discourage smoking. Majority of youth identified family factor to be most influential (64%), followed by school education (55.6%). On the contrary, youth were less receptive toward the use of anti-smoking advertisements (19.1%) and smoking alternatives (11.5%) to discourage smoking.

**Figure 2:**
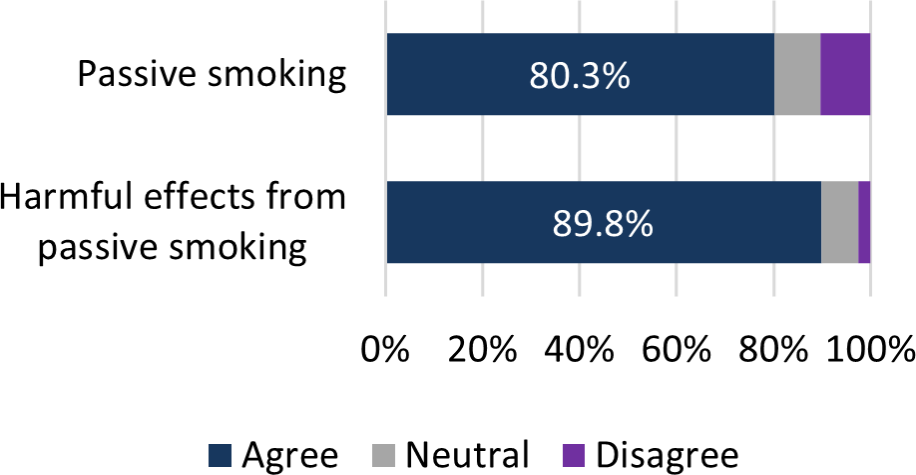
Awareness on passive smoking.

**Figure 3:**
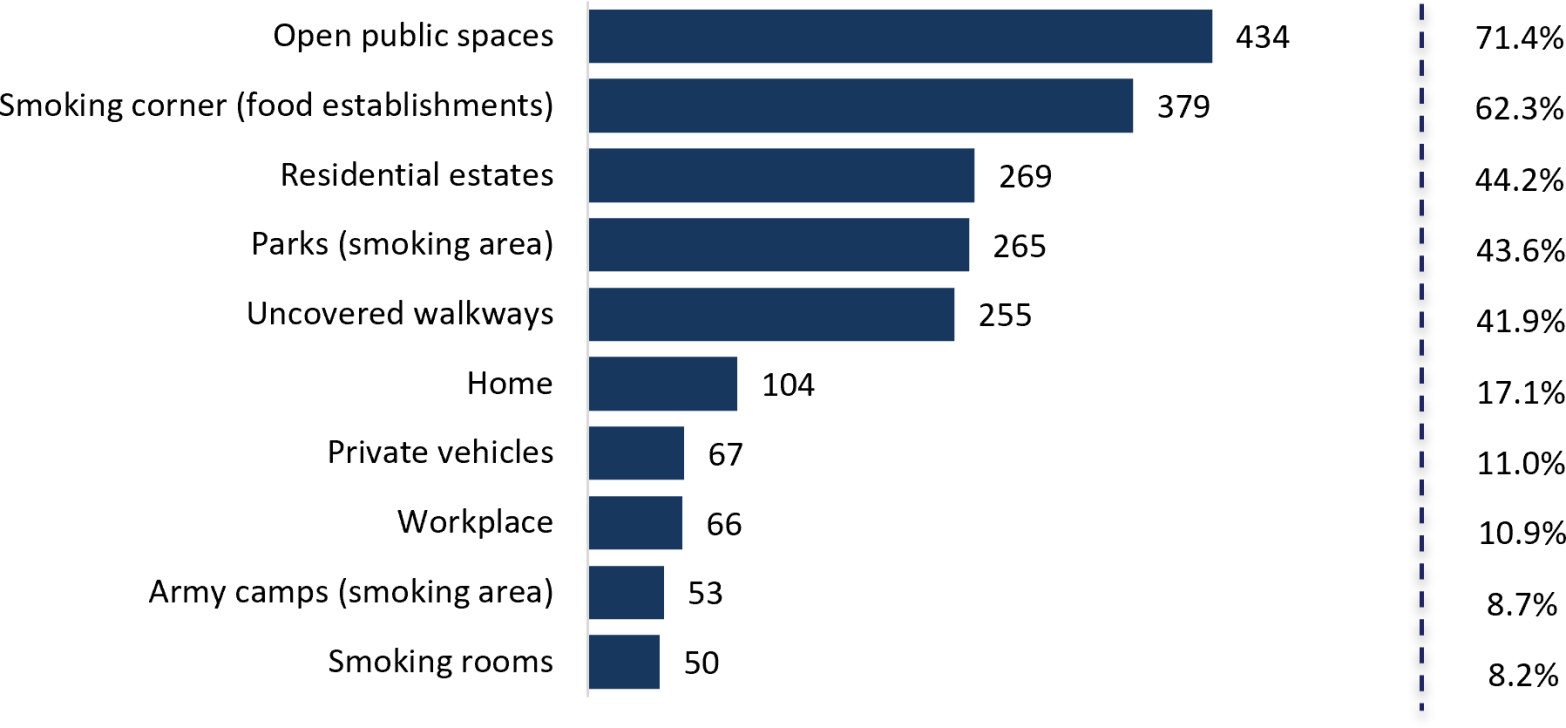
Sources of passive smoking among youth.

Figures 2, 3 shows youths’ awareness on passive smoking and its source. Overall, youth had high awareness of passive smoking (80.3%) and its harmful effects (89.8%). The most frequent source of passive smoking were open public spaces (71.4%) and smoking corners at food establishment venues (62.3%).

## 4 Discussion

This cross-sectional study explored the views of youth in Singapore on raising the MLA of smoking to 21 years old, alongside assessing their awareness on passive smoking. The study revealed that 80% of the participants supported increasing the MLA to 21 years as this policy can effectively deter youth from taking up smoking. This stands out more than the International Tobacco Control (ITC) Four Country Smoking and Vaping Survey (66%) and the US youth study by Dai on attitude regarding Tobacco 21 among US youth (63.9%) (11, 27). The strong support for raising the MLA of smoking is consistent with the strong Singaporean sentiment against smoking. Smoking has been prohibited in many public places in Singapore since 1970 with enactment and enforcement of the Smoking (Prohibition in Certain Places) Act (30). The government has progressively expanded smoking restrictions alongside restriction of cigarette promotion. However, limited studies have attempted to understand the views of youth. Their views matter as there is a risk of the *forbidden fruit effect* which is seen with legislative actions that limit access to commodities such as cigarettes, with resultant increasing curiosity to seek for cigarettes.

The study found a statistically significant association between acceptance of the higher MLA and the age of participants. Specifically, adolescents (15–17 years) were more likely to support increasing MLA to 21 years than those aged 21–24 years old. The American youth study, on the other hand, reported that support for Tobacco 21 followed a U-shaped curve according to age, with older adolescents (15–17 years) and young adults (18–20 years) having lower support rates than do younger adolescents (9–14 years) and older adults (≥21 years) (27). This suggests that younger individuals may be more receptive to the policy change, potentially due to increased awareness of the health risks associated with smoking through campaigns targeting school students.

Similarly, individuals who had never smoked were more likely to support this policy change. The Online California Adult Tobacco Survey (Online CATS) also reported that current e-cigarette users had lesser odds of agreeing the raising MLA to 21 years compared with never and former users (31). This indicates that those who have chosen not to smoke themselves are more motivated to advocate for measures aimed at reducing tobacco use among young people. These insights can inform targeted efforts to promote this policy change as increasing the MLA to 21 years may postpone young people from initiating smoking and lower their chances of transitioning into heavy smoker during adulthood.

Surprisingly, acceptance of the higher MLA was not significantly associated with gender and educational qualification. Even among individuals who were exposed to second-hand smoke in indoor environments, their attitudes toward raising the MLA did not significantly differ from those who were not exposed. The lack of association with indoor passive smoking implies that this specific policy may not be perceived as directly addressing the risk of indoor exposure to second-hand smoke.

In addition, the study aimed to understand the factors that were most effective in preventing smoking habits. Youth recognized the importance of being informed about the health implications of tobacco use through school education, family guidance, and the increasing costs associated with tobacco products as potent barriers for smoking. Correspondingly, a randomized clinical trial conducted in a US public high school found similar results on the effectiveness of school-based curriculum to reduce cigarette use by adolescents (32). This underscores the significance of incorporating comprehensive health education initiatives in school curriculum to equip youth with necessary skills and knowledge to make informed decisions about tobacco use (33, 34).

Similarly, the study result aligns with existing research on the effectiveness of tobacco taxation in deterring smoking initiation among youth. Economic studies have indicated that for every 10% increase in the real price of tobacco products, there is a 3 to 5% decrease in overall tobacco consumption, including a 3.5% reduction in young people taking up smoking (5). This demonstrates the powerful impact of economic measures, such as tobacco taxation, in curbing smoking habits among youth (35–39). However, it is important to note that the effectiveness of tobacco taxation may diminish over time due to factors such as inflation and rising incomes, underscoring the need for continuous review and adjustment of tobacco tax rates by governmental authorities to maintain their deterrent effect on smoking initiation.

While anti-smoking advertisements and graphic cigarette packaging images are widely used prevention tools, they were ranked as less effective by participants. Likewise, these strategies appeared to have limited impact on deterring smoking among those with less nicotine dependence (40). Hence, strategies should be adapted to consider the evolving needs and perceptions of youth as they grow older. Willemsen et al. concluded that addressing youth smoking requires a multi-faceted strategy comprising education, increasing costs, advertising bans and legislative measures to have a lasting impact on reducing adolescent smoking behavior (41).

People who smoke expose those who have never smoked to passive smoking through second-hand and third-hand smoking. Singaporeans are at increased risk of exposure to passive smoking as 95% live in multi-unit residential areas where smoke easily drifts to houses in proximity (42). This is consistently seen in other densely populated cities such as New York and Seoul (43, 44). In 2019, 296 deaths linked to passive smoking were reported in Singapore (22). A significant majority of the participants (80.3%) are aware of passive smoking, and an even higher percentage (89.8%) recognize its harmful effects. Awareness about passive smoking among youth is a crucial step in reducing the harm caused by secondhand smoke and promoting healthier, smoke-free environments. In 2020, a survey revealed that 85% of Singaporeans supported a proposal to ban smoking near a window or balcony in multi-unit residential areas indicating the concern about the effects of passive smoking (45).

### 4.1 Strengths

Studies targeting youths’ awareness on MLA and passive smoking are sparse. The current study offers a quick, inexpensive way through social media platforms to reach out to large groups of youth from multi-ethnic background within a relatively short time frame. Youth tend to be internet-savvy and are often more comfortable to participate in online surveys. They can complete the e-questionnaires at their own convenience. Those who smokes do not fear of any stigma and adverse reprisal in filling up anonymized survey.

### 4.2 Limitations

Nevertheless, online survey has its limitations. Such surveys may not reach a representative sample of the population, as they are limited to individuals with internet access and willingness to participate, potentially leading to selection bias. Individuals without internet access or those who are less tech-savvy might be excluded from the study but mobile phone utility among youth is prevalent in Singapore. It is difficult to decipher accurate responses from any survey because of potential social desirability among participants. In addition, the cross-sectional nature of this study does not allow for the assessment of cause-and-effect relationships or changes over time.

## 5 Conclusion

Most local Asian youth were supportive of MLA 21 and were aware of the perils of passive smoking. Only a small proportion of the participants had smoked. Coordinated efforts should be directed at the remaining youth who are less cognizant of MLA 21 and passive smoking via public education targeting at families and school campaigns against smoking. Educated youth may also influence their peers to quit the habit and contribute toward coordinated and integrated efforts to eradicate smoking for their future generations.

## Data availability statement

The data that support the findings of this study are available from the corresponding author upon reasonable request.

## Ethics statement

The studies involving humans were approved by the Hwa Chong Institution-Institutional Review Board. The studies were conducted in accordance with the local legislation and institutional requirements. Written informed consent to participate in this study was not required from the participants in accordance with the national legislation and the institutional requirements.

## Author contributions

KG: Writing – original draft. PS: Writing – original draft. DN: Data curation, Formal analysis, Writing – review & editing. EK: Data curation, Formal analysis, Writing – review & editing. HL: Conceptualization, Investigation, Writing – review & editing. RT: Conceptualization, Investigation, Writing – review & editing. YW: Conceptualization, Investigation, Writing – review & editing. NT: Conceptualization, Methodology, Writing – review & editing.
